# Financial outcomes after pediatric critical illness among commercially insured families

**DOI:** 10.1186/s13054-023-04493-8

**Published:** 2023-06-08

**Authors:** Erin F. Carlton, Michelle H. Moniz, John W. Scott, Hallie C. Prescott, Nora V. Becker

**Affiliations:** 1 Department of Pediatrics, University of Michigan, 1500 E. Medical Center Dr., Ann Arbor, MI 48109 USA; 2 Department of Obstetrics and Gynecology, University of Michigan, Ann Arbor, MI USA; 3 Department of Surgery, University of Michigan, Ann Arbor, MI USA; 4 Department of Internal Medicine, University of Michigan, Ann Arbor, MI USA

## Abstract

Critical illness results in subjective financial distress for families, but little is known about objective caregiver finances after a child’s pediatric intensive care unit (PICU) hospitalization. Using statewide commercial insurance claims linked to cross-sectional commercial credit data, we identified caregivers of children with PICU hospitalizations in January–June 2020 and January–June 2021. Credit data included delinquent debt, debt in collections (medical and non-medical), low credit score (< 660), and a composite of any debt or poor credit and were measured in January 2021 for all caregivers. For the 2020 cohort (“post-PICU”), credit outcomes in January 2021 were measured at least 6 months following PICU hospitalization and reflect financial status after the hospitalization. For the 2021 cohort (comparison), financial outcomes were measured prior to their child’s PICU hospitalization and therefore reflect pre-hospitalization financial status. We identified 2032 caregivers, 1017 post-PICU caregivers and 1015 comparison cohort caregivers, of which 1016 and 1014 were matched to credit data, respectively. Post-PICU caregivers had higher adjusted odds of having any delinquent debt [aOR 1.25; 95%CI 1.02–1.53; *p* = 0.03] and having a low credit score [aOR 1.29; 95%CI 1.06–1.58; *p* = 0.01]. However, there was no difference in the amount of delinquent debt or debt in collections among those with nonzero debt. Overall, 39.5% and 36.5% of post-PICU and comparator caregivers, respectively, had delinquent debt, debt in collections or poor credit. Many caregivers of critically ill children have financial debt or poor credit during hospitalization and post-discharge. However, caregivers may be at higher risk for poor financial status following their child’s critical illness.

**Supplementary Information:**

The online version contains supplementary material available at 10.1186/s13054-023-04493-8.

## Introduction

Prior survey studies indicate parents experience financial distress both during and after a child’s hospitalization [1, 2]. This financial distress can be related to both direct costs (e.g., out-of-pocket medical spending) and indirect costs (e.g., income loss) associated with hospitalization. However, little is known about objective financial status following a dependent child’s hospitalization. We aimed to assess objective financial status before and after Pediatric Intensive Care Unit (PICU) hospitalization.

## Methods

We used a novel dataset which linked longitudinal statewide commercial insurance claims data (Blue Cross Blue Shield of Michigan’s [BCBSM] preferred provider organization network) to cross-sectional commercial credit data (Experian) to examine financial status among caregivers of children hospitalized in the PICU. Data were accessed via the Michigan Value Collaborative (MVC), a partnership between Michigan hospitals and BCBSM [3]. MVC linked all individuals enrolled in the BCBSM network in January 2021 to their Experian commercial credit report data from January 2021 using direct patient identifiers (patient name, address, date of birth, and social security number). MVC then provided our study team with a limited use dataset without any direct patient identifiers (name, address, SSN), and all subsequent analyses were conducted using a de-identified unique person ID created specifically for the data linkage that could not be linked back to any direct patient identifiers. This project was reviewed and approved by the University of Michigan Institutional Review Board with a waiver of patient informed consent.

Using validated revenue codes [4], we identified PICU hospitalizations (age ≤ 18 years) at two time-periods: January–June 2020 and January–June 2021. Birth-related and perinatal hospitalizations were excluded. Caregivers of children hospitalized in the PICU were identified as the adults listed as the primary plan holder or spouse of the primary plan holder. Financial status of all caregivers was measured using the single observation of credit outcomes in January 2021. For the 2020 cohort, credit outcomes in January 2021 were therefore measured at least 6 months following PICU hospitalization and reflect financial status post-hospitalization. We chose this time period because there is a minimum 180 day waiting period before medical debt appears on credit reports [5]. For the 2021 cohort, financial outcomes were measured prior to their child’s PICU hospitalization and therefore reflect financial status pre-hospitalization. Going forward, we refer to the 2020 cohort as the post-PICU cohort and the 2021 cohort as the comparison cohort.

Financial outcomes included any delinquent debt (i.e., past-due debt or debt in collections), any debt in collections by type (medical and non-medical), low credit score (< 660), and a composite of any of these measures. Low credit was defined as < 660 because scores below this threshold are considered low prime or subprime and may result in higher interest rates, fees, or being denied credit altogether [6, 7]. We also measured the mean debt by category caregivers with nonzero debt in each category.

Logistic and generalized linear regression models were used to compare caregiver financial status between cohorts. All models were adjusted for age of the child, presence of a complex chronic condition [8], PICU hospitalization length of stay; age of caregiver; gender of caregiver; household status (primary plan holder with a spouse also present on the plan; primary plan holder without a spouse; and spouse) and county-level social vulnerability index quartile [9].

## Results

We identified 1017 caregivers for 589 children in the post-PICU cohort (hospitalized January–June 2020) and 1015 caregivers for 594 children in the comparison cohort (hospitalized from January–June 2021). The median age of the 1183 critically ill children in our cohort was 10.9 years (interquartile range (IQR) 3.4–15.7), 54.9% (*n* = 649) had a complex chronic condition, and median length of stay was 3 days (IQR 2–6). The top two admission diagnoses were diabetic ketoacidosis and acute respiratory failure for both cohorts, while sepsis and COVID-19 were the third most common in the post-PICU and comparison cohorts, respectively. Patient and caregiver demographics are shown in Table 1 and eTable 1.  99.9% (*n* = 2030) of caregivers were linked to their credit report data, including 1016 post-PICU caregivers whose credit report data was obtained after their child’s PICU hospitalization, and 1014 comparison caregivers whose credit report data were obtained prior to their child’s PICU hospitalization.

**Table 1:** Caregiver financial status post-PICU hospitalization

	Comparison cohort^a^	Post-PICU cohort^b^
*Characteristics of child*		
Gender		
Male	316 (53.2)	315 (53.5)
Female	277 (46.6)	271 (46.0)
Unknown	1 (0.2)	2 (0.3)
Missing/Inconsistent	< 10 (< 1.7%)	< 10 (< 1.7)
Age, years, median (IQR)	< 10 (< 1.7)	< 10 (< 1.7)
Age		
< 1 year	48 (8.1)	89 (15.1)
1–4 years	103 (17.3)	119 (20.2)
5–8 years	78 (13.1)	85 (14.4)
9–12 years	87 (14.7)	76 (12.9)
13–18 years	278 (46.8)	220 (37.4)
Length of stay, median days (95% CI)	3 (2–6)	3 (2–6)
Presence of complex chronic condition	334 (56.2)	315 (53.5)
*Characteristics of caregiver*		
Gender		
Male	495 (48.8)	504 (49.6)
Female	518 (51.0)	507 (49.9)
Unknown	< 10 (< 1.0)	< 10 (< 1.0)
Missing/Inconsistent	< 10 (< 1.0)	< 10 (< 1.0)
Age, years, median (IQR)	41.1 (35.2–46.8)	41.0 (35.1–47.0)
Age category, *n* (%)		
< 30 year	59 (5.8)	65 (6.4)
30–39 years	401 (39.5)	413 (40.6)
40–49 years	397 (39.1)	389 (38.3)
50–59 years	138 (13.6)	134 (13.2)
> = 60 years	20 (2.0)	16 (1.6)
Household status		
Primary plan holder with a spouse, *n* (%)	421 (41.5)	430 (42.3)
Spouse, *n* (%)	423 (41.7)	431 (42.4)
Primary plan holder without a spouse, *n* (%)	171 (16.9)	156 (15.3)
*Characteristics of household*^*c*^		
Social vulnerability index, *n* (%)		
0–0.25	191 (31.2)	176 (29.9)
0.25–0.50	135 (22.7)	138 (23.4)
0.50–0.75	191 (32.2)	185 (31.4)
0.75–1.0	77 (13.0)	88 (14.9)
Gini index	0.42 (0.40–0.45)	0.42 (0.39–0.45)
Median household income^d^, $ (95% CI)	65,599 (52,693–82,973)	64,420 (50,423–80,022)
Distance to PICU, miles (95% CI)	12.5 (5.8–20.4)	12.4 (6.4–20.6)
*Credit outcomes*^*e*^*, n (%)*		
Any delinquent debt	288 (28.4)	308 (30.1)
Any medical debt in collections	180 (17.8)	195 (19.2)
Any non-medical debt in collections	164 (16.2)	165 (16.2)
Low credit score	309 (30.5)	336 (33.1)
*Adjusted amount of debt*^*f,g*^		
Amount of debt due	$2910 (2316–3504)	$3424 (2700–4148)
Amount of medical debt in collections	$1213 (926–1500)	$1220 (973–1467)
Amount of non-medical debt in collections	$1997 (1626–2369)	$2126 (1736–2515)
*Adjusted odds ratio of credit outcomes*^*g,h*^		
Any delinquent debt	1.25 (1.02–1.53)	
Medical debt	1.23 (0.98–1.56)	
Non-medical debt	1.12 (0.87–1.43)	
Low credit score (< = 660)	1.29 (1.06–1.58)	

^a^Credit data measured at or before the time of PICU hospitalization

^b^Credit data measured at 6–12 months following PICU hospitalization

^c^Characteristics of the household are measured at the child level

^d^Median Household Income for patient’s ZIPCODE

^e^Unadjusted proportion of credit outcomes for each caregiver

^f^For those with nonzero debt, data indicate dollar amount (95% confidence interval)

^g^Data presented with 95% confidence intervals; adjusted for age group of the child (< 1 year; 1–4 years; 5–8 years; 9–12 years; 13–18 years, presence of a complex chronic condition, PICU hospitalization length of stay (< 3 days, 3–7 days; 7–14 days; 15–30 days; > 30 days); age group of caregiver (< 30 years; 30–39 years; 40–49 years; 50–59 years; 60 + years); gender of caregiver (male, female, unknown); household status (i.e., primary plan holder, spouse present on the plan; primary plan holder, no spouse; and spouse); quartile of social vulnerability index

^h^Odds ratios for any delinquent debt; any medical debt in collections; and any non-medical debt in collections. Reference category is the comparison group. Complete regression analysis, including odds ratios for all variables, is available in the Additional file 1: eTables 2–5

**Fig. 1:**
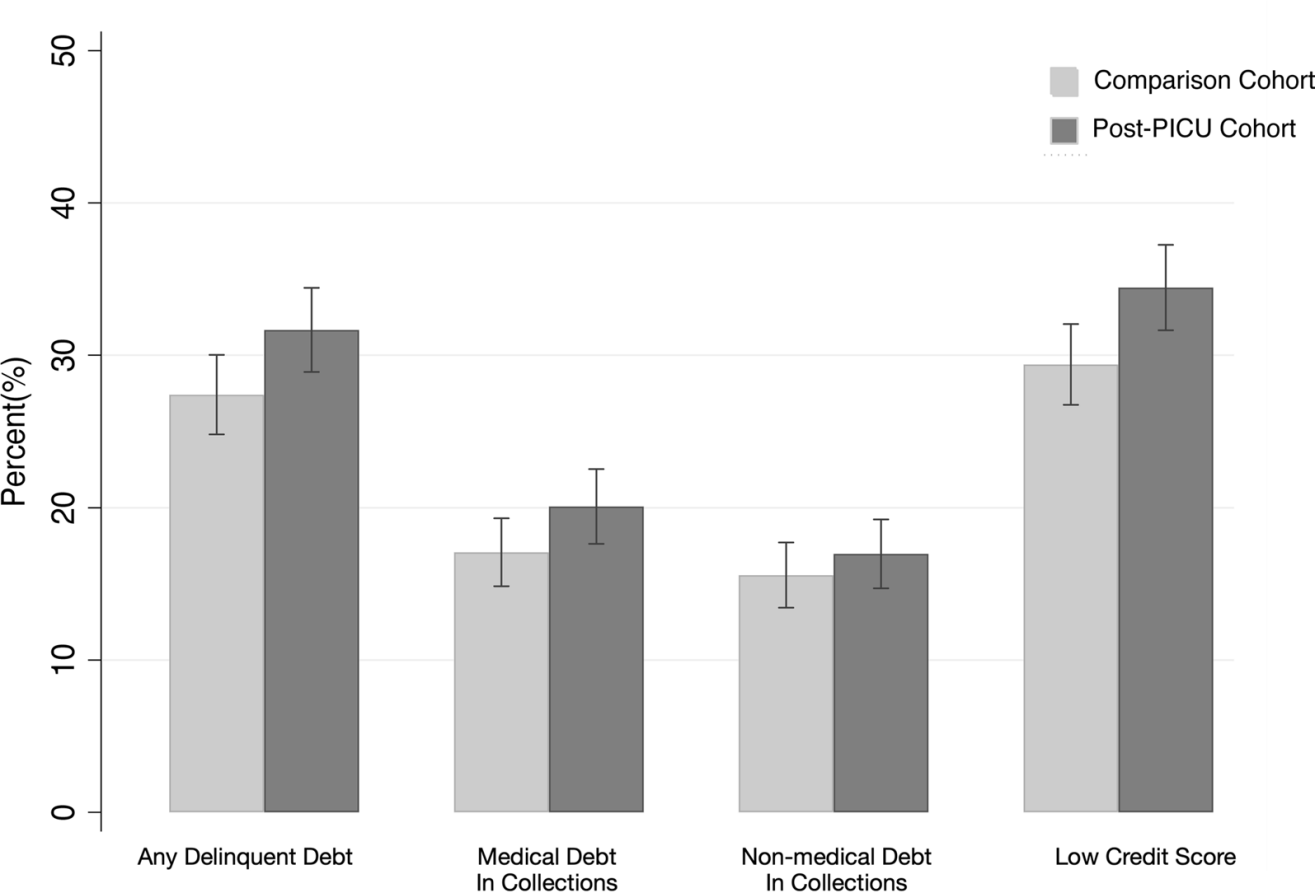
Adjusted proportion of caregivers’ financial measures before and after their child’s PICU hospitalization. This figure displays the adjusted rates of any delinquent debt, medical debt in collections, non-medical debt in collections, and low credit score by cohort. The adjusted rates displayed were obtained via logistic regression adjusting for age group of the child, presence of a complex chronic condition for the hospitalized child, PICU hospitalization length of stay, age group of the caregiver, gender of the caregiver, household status, and county-level social vulnerability index quartile. Post-PICU caregivers had a significantly higher adjusted rate of delinquent debt and low credit score compared to the comparison cohort. There was no statistically significant difference in medical and non-medical debt in collection between cohorts

Post-PICU caregivers had higher adjusted odds of having any delinquent debt [adjusted odds ratio (aOR) 1.25; 95% CI 1.02–1.53; *p* = 0.03] or having a low credit score [aOR 1.29; 95%CI 1.06–1.58; *p* = 0.01] versus the comparison group (Table 1). The post-PICU caregivers also had higher frequency of medical debt in collections and non-medical debt in collections than comparison caregivers, but the differences were not statistically significant (Fig. 1). Among caregivers with nonzero debt outcomes, there was no significant difference in the adjusted mean amounts of delinquent debt, medical debt in collections, or non-medical debt in collections (Table 1). The unadjusted distribution of debt among caregivers with non-zero debt is shown in Additional File 1: eFigures 1–3. Overall, 39.5% and 36.5% of post-PICU and comparator PICU caregivers had any delinquent debt, debt in collections or poor credit, respectively. Post-PICU caregivers had a significantly higher odds of having the composite outcome of any delinquent debt, debt in collections or poor credit (aOR 1.31; 95% CI 1.08–1.58; *p* = 0.01).

## Discussion

In this cohort of commercially insured caregivers of children hospitalized for critical illness, financial measures were worse among post-PICU caregivers compared to caregivers whose financial outcomes were measured prior to their child’s hospitalization. Post-PICU caregivers had a higher odds of having any delinquent debt or having a low credit score. However, there was no difference in the amount of debt among individuals with nonzero debt outcomes between cohorts.

Measures of debt or poor credit were common and present in over one-third of caregivers both before and after PICU hospitalization. Socioeconomic status, which includes family financial status, has been associated with higher rates of PICU hospitalization and worse outcomes [10, 11]. However, our study is among the first to directly examine objective measures of caregiver financial status before and after a child’s critical illness. Given the high rates of debt and poor credit in the comparison group, consideration should be given to screening for financial hardship or stress during hospitalization and offering financial counseling, as many parents want to discuss cost during their child’s hospitalization [12, 13].

Our results suggest that caregivers’ financial status was worse after PICU hospitalization compared with before, as the odds of having delinquent debt or a low credit score were higher in the post-PICU cohort. Furthermore, the magnitude of debt owed was substantial following PICU hospitalization, as mean debt for those with any debt was nearly $3500. Given that prior research has shown that 1 in 4 families has less than $400 in liquid assets [14], this is an amount nearly nine times than what many families can afford. Additionally, 1 in 3 caregivers had a low credit score following their child’s PICU hospitalization. Low credit scores can lead to difficulty in not only obtaining loans or applying for a mortgage, but also renting an apartment, applying for a car loan, and paying utilities [6]. Thus, a decline in credit score may be far-reaching and could potentially impact a child’s subsequent health outcomes.

While our findings indicate caregivers may face poor financial status following their child’s critical illness, the drivers of this increased odds of debt or poor credit are unknown. It is possible that the direct costs of health care related to the hospitalization are at play. Indeed, a recent study of commercially insured children found out-of-pocket spending averaged $1300 for pediatric hospitalizations, with 1 in 7 totaling over $3000 [15]. Additionally, there are often ongoing health care costs following PICU discharge [16]. Non-medical costs (*e.g.*, direct costs of meals or travel and indirect costs of lost work) may also be a key driver of debt and poor credit following PICU hospitalization [2, 13, 16]. Prior work suggests that the indirect financial costs of critical illness may be large, as 4 in 5 caregivers miss a median of 2 weeks of work during their child’s PICU hospitalization [17]. Thus, one potential mechanism of financial hardship may include missed work by caregivers which causes decreased income. This loss of income may cause families to acquire increased non-medical debt and ultimately worsened credit scores, as observed in our study. Further evaluation is needed to identify and ultimately intervene on these drivers of poor financial health.

This study has several limitations. Credit outcomes were available only for a single time-point, so pre- and post-hospitalization credit outcomes were measured in separate cohorts. Thus, there may be unmeasured differences in between the cohort including patient characteristics, hospitalization characteristics, and patient-level socioeconomic factors. However, the observed characteristics were similar. Second, we cannot rule out the presence of reverse-causality (i.e., worsening financial status could increase risk of critical illness) [10]. Third, the data come from a single-state, employer-based, commercially insured population and our cohort is older than other previously reported PICU cohorts [18], limiting generalizability. Fourth, financial status may have been improved across the board by pandemic-related payments [19]. Finally, as unemployment increased during the early pandemic [20], the generalizability may be further limited as the cohort had at least one employed caregiver (Additional file 1).

In this cross-sectional study, caregivers of critically ill children were found to have delinquent debt or poor credit both at the time of their child’s hospitalization and post-discharge. Our results suggest that caregiver financial status may be worse following their child’s hospitalization for critical illness. Further research with longitudinal assessment of families before and after critical illness is needed to better assess the financial impact of pediatric critical illness.

## Supplementary Information

**Additional file 1. Supplemental Figure 1.** Distribution of Delinquent Debt Among Caregivers With Non-Zero Debt. **Supplemental Figure 2.** Distribution of Medical Debt in Collections Among Caregivers With Non-Zero Debt. **Supplemental Figure 3.** Distribution of Non-Medical Debt in Collections Among Caregivers With Non-Zero Debt. **eTable 1.** Distribution of PICU patients by ZIP Code. **eTable 2.** Logistic Regression, Any Delinquent Debt. **eTable 3.** Logistic Regression, Any Medical Debt in Collections. **eTable 4.** Logistic Regression, Any Non-Medical Debt in Collections. **eTable 5.** Logistic Regression, Low Credit Score.

## Data Availability

Not applicable.
