# Supplementary material for: Financial outcomes after pediatric critical illness among commercially insured families
Source: Crit Care. 2023 Jun 8;27:227. doi: 10.1186/s13054-023-04493-8 (PMC10249539; doi:10.1186/s13054-023-04493-8)
Supplement: Supplementary file 1 — Additional file 1. Supplemental Figure 1. Distribution of Delinquent Debt Among Caregivers With Non-Zero Debt. Supplemental Figure 2. Distribution of Medical Debt in Collections Among Caregivers With Non-Zero Debt. Supplemental Figure 3. Distribution of Non-Medical Debt in Collections Among Caregivers With Non-Zero Debt. eTable 1. Distribution of PICU patients by ZIP Code. eTable 2. Logistic Regression, Any Delinquent Debt. eTable 3. Logistic Regression, Any Medical Debt in Collections. eTable 4. Logistic Regression, Any Non-Medical Debt in Collections. eTable 5. Logistic Regression, Low Credit Score. [file 13054_2023_4493_MOESM1_ESM.docx]

**Financial Outcomes After Pediatric Critical Illness Among Commercially Insured Families**

Supplemental Material

Erin F. Carlton, MD, MSc^1^, Michelle H. Moniz, MD, MSc^2^, John W. Scott, MD, MPH^3^, Hallie C. Prescott MD, MSc^4^, Nora V. Becker, MD, PhD^4^

Supplemental Figure 1. Distribution of Delinquent Debt Among Caregivers With Page 3

Non-Zero Debt

Supplemental Figure 2. Distribution of Medical Debt in Collections Among Page 4

Caregivers With Non-Zero Debt

Supplemental Figure 3. Distribution of Non-Medical Debt in Collections Among Page 5

Caregivers With Non-Zero Debt

eTable 1. Distribution of PICU patients by ZIP Code Page 6

eTable 2 Logistic Regression, Any Delinquent Debt Page 7

eTable 3 Logistic Regression, Any Medical Debt in Collections Page 8

eTable 4 Logistic Regression, Any Non-Medical Debt in Collections Page 9

eTable 5 Logistic Regression, Low Credit Score Page 10

**Supplemental Figure 1. Distribution of Delinquent Debt Amount Among Caregivers with Non-Zero Debt.** Overall, 289 of 1014 caregivers in the comparison cohort and 308 of 1016 in the post-PICU cohort had non-zero delinquent debt.

**
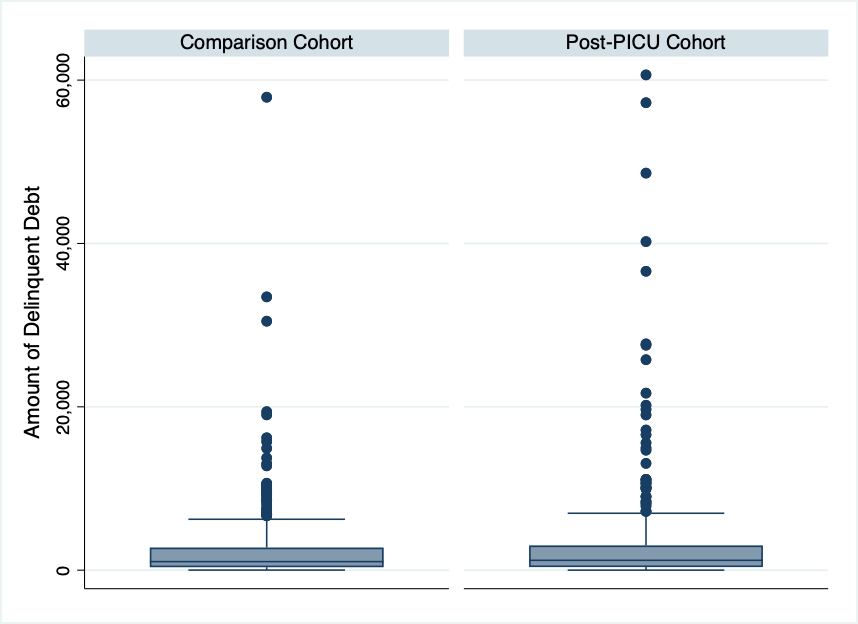
**

**Supplemental Figure 2. Distribution of Medical Debt in Collections Amount Among Caregivers with Non-Zero Debt.** Overall, 180 of 1014 caregivers in the comparison cohort and 195 of 1016 in the post-PICU cohort had non-zero medical debt in collections.


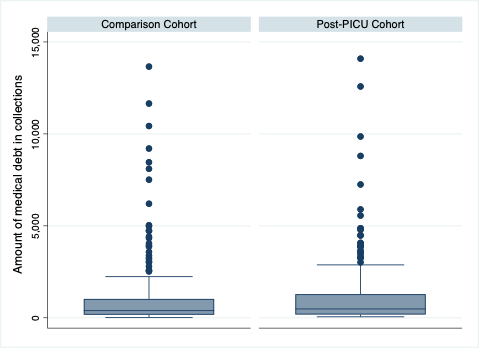


**Supplemental Figure 3. Distribution of Non-Medical Debt in Collections Amount Among Caregivers with Non-Zero Debt.** Overall, 164 of 1014 caregivers in the comparison cohort and 165 of 1016 in the post-PICU cohort had non-zero non-medical debt in collections.


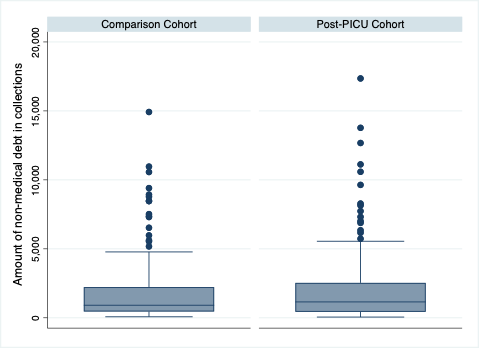


eTable 1. Distribution of PICU patients by ZIP Code

|  | Comparison (n=594) | Post-PICU(n=589) |
| --- | --- | --- |
| Detroit | 200 (33.7) | 185 (31.4) |
| Flint | 32 (5.4) | 49 (8.3) |
| Saginaw | 35 (5.9) | 26 (4.4) |
| Lansing | 39 (6.6) | 53 (9.0) |
| Kalamazoo | 29 (4.9) | 20 (3.4) |
| South Bend | <10 (<1.7) | <10 (<1.7) |
| Jackson | 11 (1.9) | 13 (2.2) |
| Grand Rapids | 35 (5.9) | 37 (6.3) |
| Cadillac | <10 (<1.7) | <10 (<1.7) |
| Mackinaw City | <10 (<1.7) | 14 (2.4) |
| Iron Mountain | <10 (<1.7) | <10 (<1.7) |
| Out-of-state | 194 (32.7) | 180 (30.6) |

eTable 2. Logistic Regression, Any Delinquent Debt

|  | Odds Ratio | 95% Confidence Interval | |
| --- | --- | --- | --- |
| **Post-PICU** | 1.252 | 1.022 | 1.533 |
| **Child Age Category** |  |  |  |
| < 1 year | Ref |  |  |
| 1-4 years | 1.109 | .744 | 1.654 |
| 5-8 years | 1.966 | 1.279 | 3.023 |
| 9-12 years | 2.585 | 1.661 | 4.022 |
| 13-18 years | 3.386 | 2.238 | 5.124 |
| **Parent Age Category** |  |  |  |
| < 30 years | Ref |  |  |
| 30-39 years | .557 | .362 | .856 |
| 40-49 years | .289 | .177 | .471 |
| 50-59 years | .207 | .118 | .363 |
| >=60 years | .147 | .05 | .432 |
| **Length of Stay Category** |  |  |  |
| < 3 days | Ref |  |  |
| 3-6 days | 1.045 | .832 | 1.313 |
| 7-14 days | .911 | .657 | 1.262 |
| 15-29 days | 1.275 | .82 | 1.981 |
| 30+ days | .595 | .254 | 1.392 |
| **Gender of Caregiver** |  |  | . |
| Female | 1.067 | .862 | 1.322 |
| Unknown | 1 | . | . |
| **Complex Chronic Condition (child)** | .949 | .769 | 1.172 |
| **Household Status** |  |  |  |
| Primary plan holder with a spouse | 1 | . | . |
| Spouse | 1.094 | .863 | 1.387 |
| Primary plan holder without a spouse | 2.305 | 1.736 | 3.061 |
| **Social Vulnerability Index** |  |  |  |
| 0-0.25 | 1 | . | . |
| 0.25-0.50 | 1.4 | 1.046 | 1.873 |
| 0.50-0.75 | 2.108 | 1.624 | 2.735 |
| 0.75-1.0 | 2.069 | 1.503 | 2.848 |

eTable 3. Logistic Regression, Any Medical Debt in Collections

|  | Odds Ratio | 95% Confidence Interval | |
| --- | --- | --- | --- |
| **Post-PICU** | 1.234 | .978 | 1.558 |
| **Child Age Category** |  |  |  |
| < 1 year | Ref |  |  |
| 1-4 years | .924 | .583 | 1.467 |
| 5-8 years | 1.692 | 1.032 | 2.773 |
| 9-12 years | 1.747 | 1.049 | 2.911 |
| 13-18 years | 2.428 | 1.51 | 3.903 |
| **Parent Age Category** |  | . | . |
| < 30 years | Ref |  |  |
| 30-39 years | .659 | .403 | 1.077 |
| 40-49 years | .35 | .202 | .607 |
| 50-59 years | .3 | .158 | .571 |
| >=60 years | .233 | .068 | .806 |
| **Length of Stay Category** |  | . | . |
| < 3 days | Ref |  |  |
| 3-6 days | 1.101 | .847 | 1.431 |
| 7-14 days | .867 | .593 | 1.269 |
| 15-29 days | 1.099 | .661 | 1.83 |
| 30+ days | .221 | .051 | .955 |
| **Gender of Caregiver** |  | . | . |
| Female | 1.321 | 1.035 | 1.686 |
| Unknown | 1 | . | . |
| **Complex Chronic Condition (child)** | 1.122 | .881 | 1.43 |
| **Household Status** |  |  |  |
| Primary plan holder with a spouse | Ref |  |  |
| Spouse | 1.749 | 1.255 | 2.437 |
| Primary plan holder without a spouse | 1.143 | .87 | 1.5 |
| **Social Vulnerability Index** |  |  |  |
| 0-0.25 | Ref |  |  |
| 0.25-0.50 | 1.76 | 1.258 | 2.463 |
| 0.50-0.75 | 2.141 | 1.571 | 2.917 |
| 0.75-1.0 | 1.631 | 1.106 | 2.404 |

eTable 4. Logistic Regression; Any non-medical debt in collections

|  | Odds Ratio | 95% Confidence Interval | |
| --- | --- | --- | --- |
| **Post-PICU** | 1.117 | .871 | 1.432 |
| **Child Age Category** |  |  |  |
| < 1 year | ref | . | . |
| 1-4 years | 1.648 | .974 | 2.787 |
| 5-8 years | 2.692 | 1.529 | 4.74 |
| 9-12 years | 3.18 | 1.782 | 5.675 |
| 13-18 years | 3.398 | 1.941 | 5.947 |
| **Parent Age Category** |  |  |  |
| < 30 years | Ref | . | . |
| 30-39 years | .518 | .318 | .846 |
| 40-49 years | .288 | .165 | .503 |
| 50-59 years | .204 | .105 | .396 |
| >=60 years | .217 | .063 | .75 |
| **Length of Stay Category** |  |  |  |
| < 3 days | Ref | . | . |
| 3-6 days | 1.197 | .908 | 1.578 |
| 7-14 days | .865 | .567 | 1.32 |
| 15-29 days | 1.157 | .664 | 2.016 |
| 30+ days | .983 | .352 | 2.749 |
| **Gender of Caregiver** | Ref | . | . |
| Female | .868 | .665 | 1.132 |
| Unknown | 1 | . | . |
| **Complex Chronic Condition (child)** | .935 | .721 | 1.213 |
| **Household Status** |  |  |  |
| Primary plan holder with a spouse | Ref | . | . |
| Spouse | 2.95 | 2.127 | 4.09 |
| Primary plan holder without a spouse | 1.184 | .87 | 1.611 |
| **Social Vulnerability Index** |  |  |  |
| 0-0.25 | Ref | . | . |
| 0.25-0.50 | 1.091 | .745 | 1.596 |
| 0.50-0.75 | 1.78 | 1.292 | 2.451 |
| 0.75-1.0 | 1.975 | 1.352 | 2.884 |

eTable 5. Logistic regression; Low credit score

|  | Odds Ratio | 95% Confidence Interval | |
| --- | --- | --- | --- |
| **Post-PICU** | 1.2948 | 1.061 | 1.58 |
| **Child Age Category** |  |  |  |
| < 1 year | Ref | . | . |
| 1-4 years | 1.45 | .972 | 2.164 |
| 5-8 years | 2.289 | 1.483 | 3.532 |
| 9-12 years | 3.134 | 2.016 | 4.874 |
| 13-18 years | 3.986 | 2.626 | 6.051 |
| **Parent Age Category** |  |  |  |
| < 30 years | Ref | . | . |
| 30-39 years | .513 | .337 | .783 |
| 40-49 years | .273 | .17 | .44 |
| 50-59 years | .142 | .081 | .249 |
| >=60 years | .201 | .077 | .528 |
| **Length of Stay Category** |  |  |  |
| < 3 days | Ref | . | . |
| 3-6 days | 1.034 | .825 | 1.294 |
| 7-14 days | .867 | .628 | 1.197 |
| 15-29 days | 1.23 | .803 | 1.884 |
| 30+ days | .887 | .404 | 1.947 |
| **Gender of Caregiver** | Ref | . | . |
| Female | 1.076 | .871 | 1.329 |
| Unknown | 1 | . | . |
| **Complex Chronic Condition (child)** | .834 | .677 | 1.026 |
| **Household Status** |  |  |  |
| Primary plan holder with a spouse | Ref | . | . |
| Spouse | 2.687 | 2.028 | 3.561 |
| Primary plan holder without a spouse | .982 | .778 | 1.24 |
| **Social Vulnerability Index** |  |  |  |
| 0-0.25 | Ref | . | . |
| 0.25-0.50 | 1.273 | .959 | 1.69 |
| 0.50-0.75 | 1.663 | 1.29 | 2.143 |
| 0.75-1.0 | 1.73 | 1.263 | 2.369 |
